# 3D Printed Reversible Shape Changing Components with Stimuli Responsive Materials

**DOI:** 10.1038/srep24761

**Published:** 2016-04-25

**Authors:** Yiqi Mao, Zhen Ding, Chao Yuan, Shigang Ai, Michael Isakov, Jiangtao Wu, Tiejun Wang, Martin L. Dunn, H. Jerry Qi

**Affiliations:** 1 The George W. Woodruff School of Mechanical Engineering, Georgia Institute of Technology, Atlanta, GA 30332, USA; 2 Singapore University of Technology and Design, Singapore 138682, Singapore; 3 State Key Lab for Strength and Vibration of Mechanical Structures, School of Aerospace Science, Xian Jiaotong University, Xian 710049, China; 4 Department of Mechanics, School of Civil Engineering, Beijing JiaoTong University, Beijing, 100044, China

## Abstract

The creation of reversibly-actuating components that alter their shapes in a controllable manner in response to environmental stimuli is a grand challenge in active materials, structures, and robotics. Here we demonstrate a new reversible shape-changing component design concept enabled by 3D printing two stimuli responsive polymers—shape memory polymers and hydrogels—in prescribed 3D architectures. This approach uses the swelling of a hydrogel as the driving force for the shape change, and the temperature-dependent modulus of a shape memory polymer to regulate the time of such shape change. Controlling the temperature and aqueous environment allows switching between two stable configurations – the structures are relatively stiff and can carry load in each – without any mechanical loading and unloading. Specific shape changing scenarios, e.g., based on bending, or twisting in prescribed directions, are enabled via the controlled interplay between the active materials and the 3D printed architectures. The physical phenomena are complex and nonintuitive, and so to help understand the interplay of geometric, material, and environmental stimuli parameters we develop 3D nonlinear finite element models. Finally, we create several 2D and 3D shape changing components that demonstrate the role of key parameters and illustrate the broad application potential of the proposed approach.

The creation of reversibly-actuating components that alter their shapes in a controllable manner in response to environmental stimuli is a grand challenge in active materials, structures, and robotics, as these components can be used as platform technologies in a wide array of applications, ranging from biomedical devices to smart packaging^1^,^2^,^3^. In these applications, large, reversible actuations, which can switch between two complicated shapes in a precisely controlled manner, are highly sought-after yet extremely challenging to achieve. For example, in smart goods applications, it is highly desirable that the packaging or labeling materials have some “intelligence” to recognize and respond to environmental changes^3^. Shape memory materials, including shape memory alloys (SMAs) and shape memory polymers (SMPs), are candidates that have been extensively explored for smart structure/device applications^4^. However, both materials have some drawbacks: SMAs have sufficient stiffness and can offer reversible actuation, but the amount of actuation is typically small^5^,^6^; SMPs are complementary in that they can offer large shape change^7^,^8^,^9^,^10^, but generally only in one-way actuation. A notable exception is that of Mather *et al*.^11^ who demonstrated two-way actuation by using a semi-crystalline polymer, where an external biasing load had to be maintained. This condition was later removed by integrating an external bias into a composite architecture, where an SMP programmed strip was embedded inside another polymer matrix^12^; more recently this concept was adopted to create interpenetrating network polymers, where one group of polymers could crystallize upon temperature change^13^. But these approaches are limited to simple shape changes because they are determined by the mechanical loading applied, which has been limited to simple tension. Environmentally responsive hydrogels^14^,^15^,^16^, on the other hand, are active materials capable of two-way actuation; they reversibly swell or shrink as environmental conditions, such as temperature or PH value, change reversibly. However, hydrogels are soft, with a Young’s modulus typically of a few tens to hundreds of kPa, limiting their applicability, e.g., for some biomedical applications. Generally the advances in the development of these active polymer materials have focused on the sophisticated multiphysics constitutive behavior of the materials, although the pursuit of composite concepts has integrated this with the control of material architecture in fibrous or layered forms.

3D printing (or additive manufacturing) is an advanced manufacturing technique that allows materials to be deposited in a layer-by-layer manner to form a 3D component. Since the printer controls the profile of each layer, components with complicated shapes (both exterior and interior geometries) can be printed relatively easily. The recent development of multi-material 3D printing techniques (such as the polyjet technique) enables printing of digital materials whose properties can vary almost continuously within a large range at almost any predefined spatial location. Such capabilities, i.e. printing complicated geometries and digital materials, empower 3D printing to be readily integrated with novel designs to create materials or components with unprecedented properties, such as metamaterials with negative Poisson’s ratio^17^, ultralight and ultrastiff metamaterials^18^, terahertz plasmonic waveguides^19^, acoustic cloaks^20^, and medical devices^21^,^22^,^23^. These impressive achievements have largely relied on the control of material architecture in 3D to achieve the desired behavior, but recently a 4D printing concept was proposed where active materials were used to create shape changing components^24^,^25^,^26^,^27^,^28^,^29^,^30^ by carefully placing them in 3D space, which added the 4^th^ dimension (or time) to the 3D printing process.

In this work, we integrated environmentally-responsive SMPs and hydrogels into 3D architectures enabled by multimaterial 3D printing to create components that can be reversibly switched between two stable and stiff configurations without the application of mechanical loading for training. We created 3D architectures that impose internal mechanical constraints from the SMPs to transform the pressure generated from equiaxial hydrogel swelling into a uniaxial driving force, which drives shape change in a prescribed way; we also use the shape memory effect in the SMP to regulate the time dependence of such shape changes. In addition, the SMP provides the component with stiffness that is much higher than one would achieve in purely hydrogel-based components. In this concept, the interactions of different materials and their spatial and temporal arrangements determine the geometrical and temporal shape change characteristics and the mechanical stiffness and load-carrying capacity in each configuration. In order to understand the operative phenomena and exploit this in design we develop a computational model that integrates the sophisticated constitutive behaviors of active materials (both the SMP and the hydrogel) with the controlled spatial distributions of these materials in 3D space to investigate the shape change behavior and to explore the design space. Finally, by applying the design principles, we create and demonstrate the behavior of several shape-changing structures that exhibit reversible shape change based on folding, curling, and origami concepts.

## Results

### Design concepts

**Figure 1:**
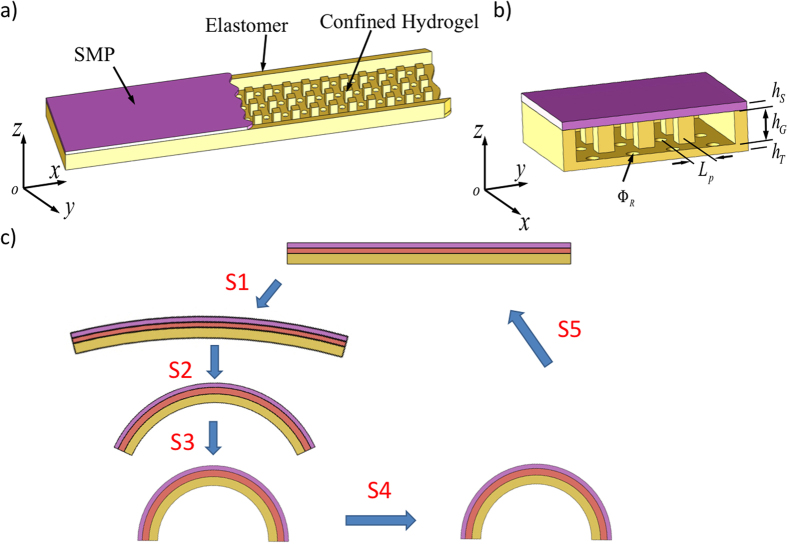
The Schematic graphs of the design concept of the two-way reversible actuator. (**a**) The reversible actuation component by 3D printing where the hydrogel is confined by the SMP and the elastomer layers. (**b**) The key dimensions in the design. (**c**) Schematic plots for a typical two way actuation: In S1, the sample is immersed in water at low temperature; in S2, it is brought to a high temperature environment and bends; in S3, it is cooled down to a low temperature; in S4, it is allowed to dry for an elongated time period; in S5, it is heated to recover the original printed shape. This finishes one actuation cycle, which can be repeated many times.

The two key concepts to our design are to convert the hydrogel’s hydraulic swelling force from equiaxial to a linear or planar force that can drive the shape change in one particular direction or one particular plane, and to use the temperature sensitivity of the SMP properties to regulate the time for such shape change. Figure 1a shows the design, where hydrogel and elastomer columns are sandwiched between a layer of the SMP (top) and a layer of the elastomer (bottom). Small holes are placed in the elastomer layer to allow water (or other solutions) to flow in and out. Figure 1b shows the notations of dimensions in the design. Figure 1c shows the flow chart of working steps for a reversible actuation cycle. The printed component is straight after printing. It is then immersed into water at a temperature ~0 °C for a certain amount of time to allow the hydrogel to absorb the water (step S1); in addition, due to the low temperature, the stiffness of the SMP is high; therefore the volume swelling of the hydrogel is highly constrained, and the strip does not show significant shape change. Next (step S2), the strip is brought into a high temperature environment (such as a water bath) where the SMP softens significantly. This reduction in the SMP stiffness allows the large shape change. In addition, the elastomer column that connects top and bottom layers imposes constraints on hydrogel swelling in the z-direction, thus converting the swelling force into the x-y plane. Because of the stiffness difference between the elastomer and the SMP, the strip bends. After actuation at high temperature, we cool the strip to a temperature below the T_g_ of the SMP (step S3), for instance, to room temperature; due to the increase of the SMP stiffness, the strip is stiff. In the ambient environment, the hydrogel loses water and dries in step S4. After the hydrogel is fully dry, heating the strip to a high temperature recovers the straight shape of the strip (step S5). At low temperature, the strip becomes stiff again. This finishes one cycle of reversible actuation, which can be repeated multiple times.

### Thermomechanical properties of printed materials

We used a multiple material 3D printer (Objet260 Connex, StrataSys, Eden Prairie, MN, USA) to realize the above concepts. The 3D printer offers a library of materials with properties ranging from rubbers to glassy polymers at room temperature (RT, 25 °C). More importantly, this printer creates so-called digital materials, which are essentially composites of the base materials tuned to have desired thermomechanical properties such that they vary in the range from rubbery to glassy. In this work, we mainly used three materials in the printer material library: Grey60, Tangoblack (TB) and a hydrogel. As shown in the Method Section, Grey60 is a digital material with the glass transition temperature (T_g_) at ~48 °C, which can be used as the SMP when the temperature is changed between 0 °C and 60 °C. TB is rubbery at RT and is used as the elastomer; the printed hydrogel absorbs water, showing a RT linear swelling ratio of 1.18, or volumetric swelling ratio of 1.64.

### Demonstration of the two-way actuation of a simple actuator

The concept described above was implemented into a simple design, which was then printed by the 3D printer. Here, the top SMP layer was 0.5 mm in thickness, and the bottom TB layer was 1.5 mm, and hydrogel layer was 0.5 mm. The radius of the hole was 0.4 mm. The pillar had a square cross-section with the edge length of 0.5 mm.

**Figure 2:**
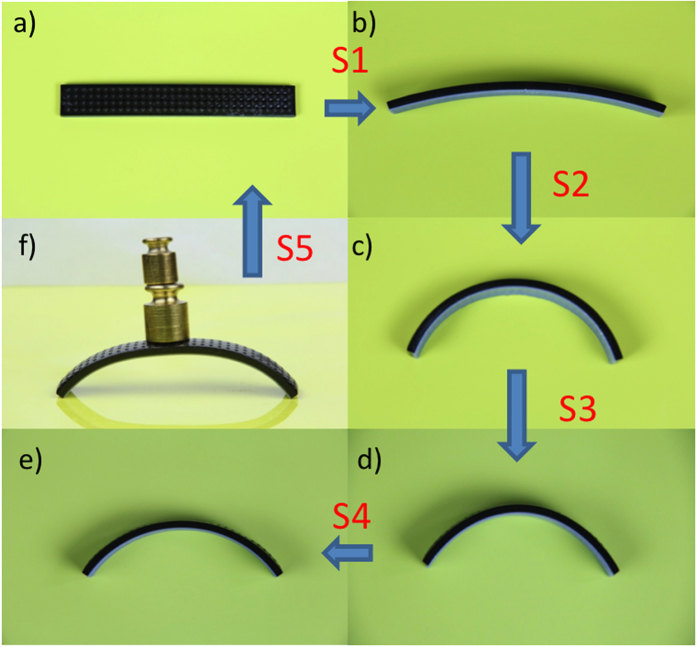
The bending angle of two activated shape memory strip in the desired shape memory cycle. (**a**) The printed strip is straight. (**b**) It bends slightly after immersing in cold water for 12 hrs. (**c**) It bends quickly when immersing in hot water. (**d**) It is then taken out of water to the room temperature air. (**e**) It is then air dried. (**f**) The strip is stiff and can carry a load of 25 g. If it is heated, it returns to the straight shape.

The printed strip is shown in Fig. 2a. It was first immersed in cold water (3 °C) for 12 hrs and showed a small amount of bending (Fig. 2b). The strip was then immersed in hot water (temperature of 75 °C) and it bent into the shape showing in Fig. 2c in ~10 sec. The strip was then removed from the water and was cooled down to RT (Fig. 2d). The strip maintained the bending shape (Fig. 2e). At the low temperature, it was stiff, which is evident in Fig. 2f where the bent strip can support a deadweight of 25 g. After drying at low temperature, the strip was immersed in high temperature water again and it recovered the flat shape.

To further examine the shape memory property, we put the sample in RT water (25 °C) again. After 12 hrs, the bending angle of the sample was larger than that in cold water (see Fig. S1a in SM). This is because the SMP have a lower modulus at RT than in cold water. When the sample was immersed in high temperature water (75 °C) again, it showed a nearly identical bending angle as that in S2. After drying in low temperature, the sample completely recovered its initial state at the high temperature. This process can be repeated multiple times.

### Parametric studies of the design

In order to gain insight on the underlying physics and the controlling design parameters for the two-way actuation, finite element method (FEM) simulations were conducted to investigate the complicated thermomechanical coupling of the SMP and the swelling property of the hydrogel by using the commercially available finite element software package ABAQUS (Dassault Systems, Johnston, RI, USA). The model details are described in the Method section. The thermomechanical properties of the elastomer and the SMP were considered using a user material subroutine based on the constitutive models developed by the authors^31^. The non-equilibrium swelling/shrinking behaviors of hydrogels were modeled by using user element (UEL) provided by Chester *et al*.^32^,^33^. To reduce the FEM model size, only a quarter of a unit cell was considered.

**Figure 3:**
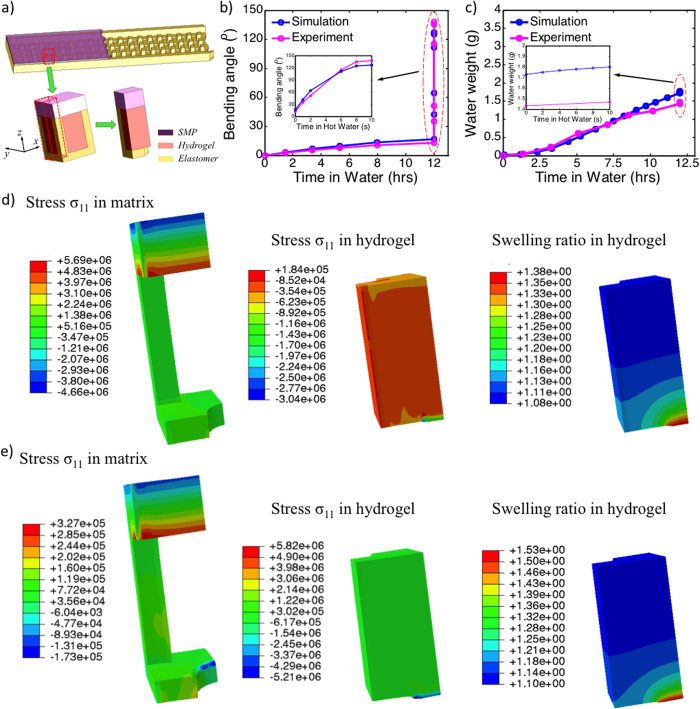
Finite element modeling of water intake and actuation. (**a**) A schematic illustration of the finite element model. (**b**) Bending angle of the strip as a function of time of the strip in water. The inset shows the bending angle when the strip is immersed in hot water. Bending angle is calculated based on a strip that is 80 mm long. (**c**) FEA simulation results of the weight of water inside the hydrogel as a function of time. Contour plots of axial stresses in the matrix and hydrogel and the swelling ratio in hydrogels at (**d**) the low temperature and (**e**) the high temperature.

Figure 3b shows the bending angle as a function of time in water. It is clear that when the strip is in the cold water, the angle evolves slowly as water is absorbed by the hydrogel. However, once the strip is in the hot water (or the high temperature environment), it bends quickly, as indicated by a sudden jump in bending angle in Fig. 3b. The inset in Fig. 3b reveals the change of angle occurs in ~10 s in hot water. Figure 3c shows the water absorbed by the hydrogel, which is a slow continuous process. Interestingly, Fig. 3c also shows that even during the sudden change of bending angle, the amount of water in-take only changes slightly, indicating that the modulus change in the SMP, which is due to the temperature increase, is the key factor for the increase in bending angles. Figure 3d shows the stress contour for the matrix (left) and the stress and swelling ratio contours for the hydrogel (middle and right) at low temperature after water intake. It can be seen that with the water intake, the SMP (top) layer develops axial stress that is featured in bending deformation (compression on the top and tension on the bottom). In addition, the swelling of hydrogel is highly non-uniform, with most swelling occurring in the area where hydrogel is in direct contact with water. This is understandable, as this area has less constraint. Figure 3e shows the same contour plots at the high temperature. In comparison with the contour at low temperature, there are two differences. First, although the trends of stress distribution in the SMP are the same, the magnitude decreases significantly. This is because of the significant decrease in the modulus of the SMP. Second, the volume of hydrogel increases. This is interesting, as Fig. 3b shows that water intake remains almost constant as we increase the temperature. Therefore, the increase of hydrogel volume is because of the decrease of hydraulic pressure and the compressible nature of a hydrogel. It should be noted that hydrogels are usually assumed to be incompressible because their shear modulus is typically much smaller than their bulk modulus. This assumption is true when the hydraulic pressure is not high. However, under the confined environment in the current study, the significant hydraulic pressure can cause observable volume change of hydrogel. To further confirm that the sudden bending of the strip is caused by the change of the modulus of the SMP, instead of water intake, we conducted experiments where we heated the sample by using an oven. The results show (in Section 2 of Supplementary Materials) that although the bending speed is slow, the strip can reach the same bending angle as that heated in hot water.

**Figure 4:**
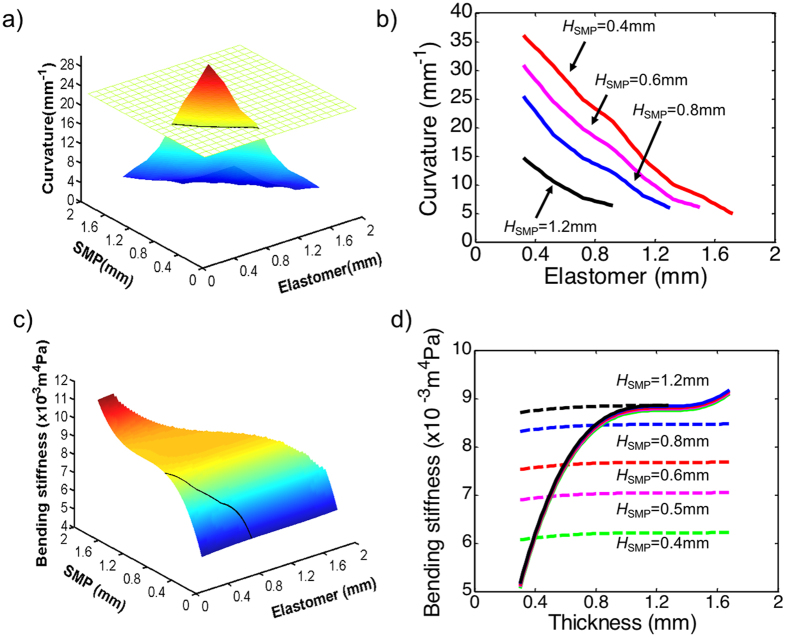
Simulation studies to explore the design space. The total thickness of the actuator strip was maintained at 2.5 mm. (**a**) A 3D plot of bending curvature as a function of the elastomer and the SMP layer thicknesses. (**b**) Bending curvature as a function of the elastomer layer thickness with different SMP layer thickness. (**c**) Bending stiffness as a function of the elastomer and the SMP layer thicknesses. (**d**) Bending stiffness as a function of the elastomer layer thickness (dash line) and SMP layer thickness (solid line).

The dimensions of different layers play a critical role in determining the bending angle of the strip. We printed and tested 6 samples with different dimensions of the elastomer, the SMP and the hydrogel layers, as shown in Table 1. It can be seen in the case of the elastomer and the SMP having the same layer thickness (0.5 mm), the thinner the hydrogel layer, the larger the bending angle change. On the other hand, when the thicknesses of the SMP and the hydrogel layers are fixed, the thinner the elastomer layer, the larger the bending angle. To further study the design space, FEM simulations were conducted. Here, in the first set to be studied, we fixed the total thickness at 2.5 mm and varied the thicknesses of the elastomer and the SMP layers (thus the hydrogel layer). Figure 4a shows a 3D plot of the curvature as a function of layer thicknesses of the elastomer and the SMP. Figure 4b shows the curvature as a function of the elastomer layer thickness with different SMP layer thicknesses. The FEM simulations confirm that decreasing elastomer thickness increases the bending curvature of the strip. The highest curvature would have been achieved if the elastomer had a zero thickness. However, this is not allowed in the real design, as the hydrogel has to be contained inside the sandwich structure enclosed by the elastomer and the SMP. Figure 4b also shows that increasing the SMP layer thickness decreases the bending curvature, which is reasonable, as the SMP has a higher modulus (even at high temperature) and thus imposes higher resistance to bending.

We also investigated the bending stiffness of the strip at low temperature. Figure 4c shows the 3D plot and Fig. 4d shows the bending stiffness with different thicknesses of the elastomer and the SMP layers. These figures show clearly that the bending stiffness is mainly determined by the SMP layer thickness. Moreover, the dependence of the bending stiffness on SMP layer thickness is nonlinear. For example, the bending stiffness is almost independent of the SMP layer thickness if it is between ~1.0 mm and ~1.6 mm. According to Fig. 4b, since the thinner SMP layer leads to higher bending curvature, using a SMP layer thickness of ~1.0 mm is beneficial, as it offers a high bending curvature as well as a high bending stiffness.

**Figure 5:**
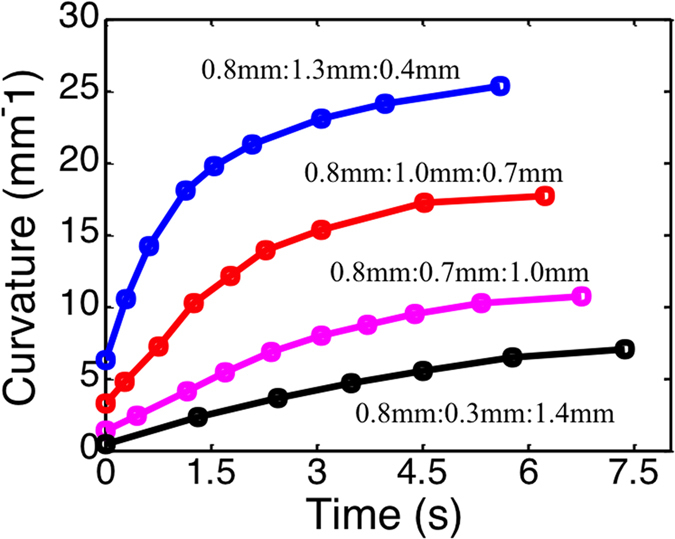
Actuation speeds of actuators with different dimensions. The change of bending curvature as a function of time for the actuators with different layer thicknesses (expressed as thicknesses of Elastomer:Hydrogel:SMP) during the S2 (heating).

Another consideration in the design is the actuation speed. FEM simulations were used to explore this design aspect. Here, although many factors, such as the times for water absorption and depletion, can affect the actuation speed, we demonstrated the cases where we varied the dimensions of the component. For all the cases shown and discussed below, we let the component absorb water at 3 °C for 12 hrs; we then took it to the high temperature of 75 °C to allow the shape change. The change of bending curvature as a function of time (measured from the time when heating starts) during heating was monitored and is reported in Fig. 5 for the components with different layer thicknesses, represented as h_Elastomer_:h_hydrogel_:h_SMP_ in the figure. Here, we varied the thickness of the SMP and the hydrogel layers but maintained the total layer thickness to be constant at 2.5 mm and the elastomer layer thickness to be 0.8 mm. It can be seen that a thinner SMP layer generally promotes a larger curvature and a faster rate of curvature change. The difference in curvatures and curvature change rates can be used to design sequential folding patterns, as will be shown later in this paper.

### 2D shape change objects

**Figure 6:**
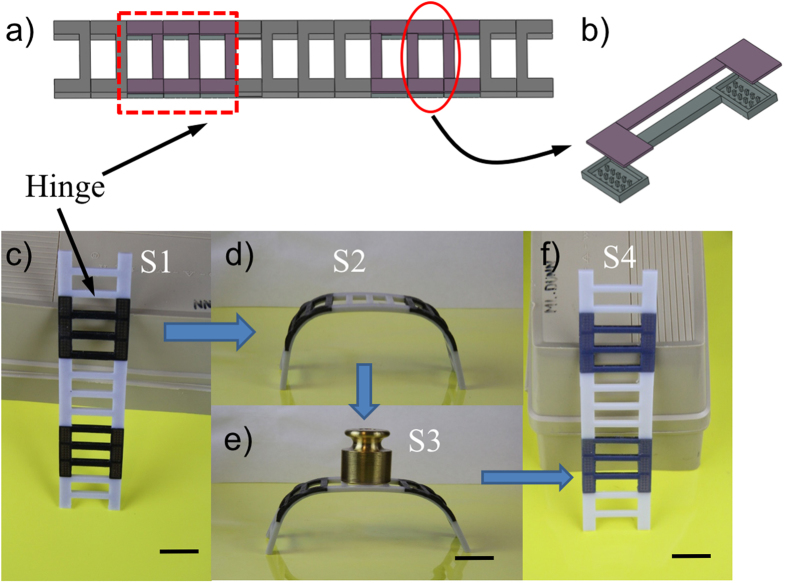
A reversible ladder and bench design. (**a**) The design of the ladder with two hinges. (**b**) each hinge contains the reversible component. (**c**) The printed ladder. (**d**) It bends into a bench shape after swelling in low temperature water for 10 hrs, followed by heating to 75 °C then cooling to the RT. (**e**) The bench is stiff and can carry a load of 50 g. (**f**) The bent shape is recovered when the bench is put in high temperature water (75 °C). The scale bars represent 20 mm.

To demonstrate the potential of our approach on reversible actuation of the design, we used the bending behaviors to create different shape changing structures. In the first example, we applied the reversible actuation concept to a hinge design, which was applied to a ladder where some portions of its structure have the reversible actuation components (Fig. 6a). The hinge of the ladder consisted of three elements discussed above, where the thicknesses of the elastomer, the hydrogel, and the SMP layers were 0.5 mm, 1.5 mm and 0.5 mm (shown in Fig. 6b), respectively. The printed ladder is shown in Fig. 6c. Similar to the general reversible activation cycle discussed in Fig. 2a, after allowing the ladder in the low temperature water (3 °C) for ~10 hrs, the hinges bent slightly. After putting it in high temperature water (75 °C), it deformed into a bench as shown in Fig. 6d. The bending configuration was fixed in low temperature (3 °C) due to the glass transition property of the SMP layer. After drying in low temperature (3 °C), the temporary configuration was sufficiently stiff and could sustain a 50 g weight as shown in Fig. 6e. After heating the bench to 75 °C, it became a straight ladder again (Fig. 6f). This process can be repeated many times.

**Figure 7:**
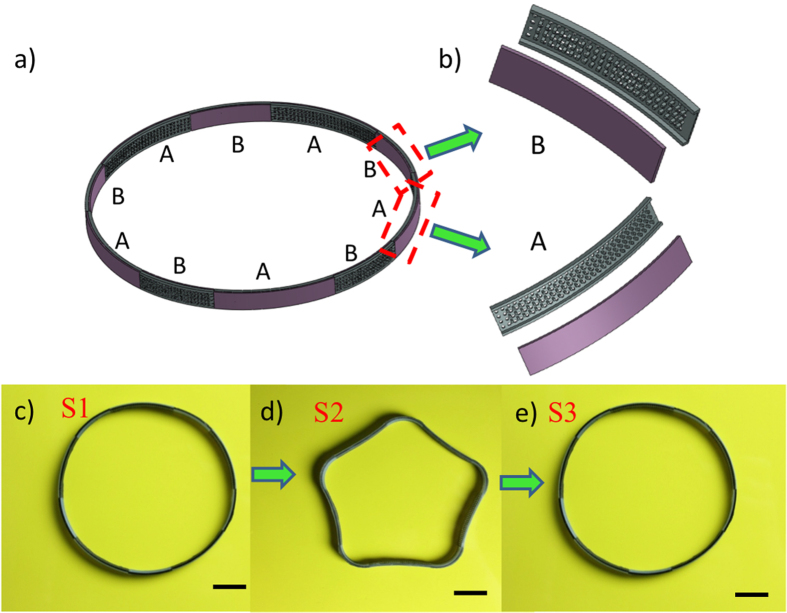
A ring that can reversibly change its shape. (**a**) The schematic graph of the reversible shape changing ring with ten A-B alternating sections. (**b**) The details of the A and B sections. (**c–e**) The reversible shape change from experimental results. The scale bars in (**c–e**) represent 15 mm.

Another example of the reversible component design is shown in Fig. 7a,b, where a ring is designed with ten A-B alternating sections. The thicknesses of the elastomer, the hydrogel and the SMP layers were 0.25 mm, 0.6 mm and 0.45 mm, respectively. When under steps similar to those discussed in Fig. 2a, the printed circular ring (Fig. 7c) transformed into a pentagram star after being immersed in cold water for 12 hrs and then placed in hot water. After drying in low temperature, the star was stiff (Fig. 7d). This configuration could be recovered to the circular ring again under the thermal activation process (Fig. 7e).

**Figure 8:**
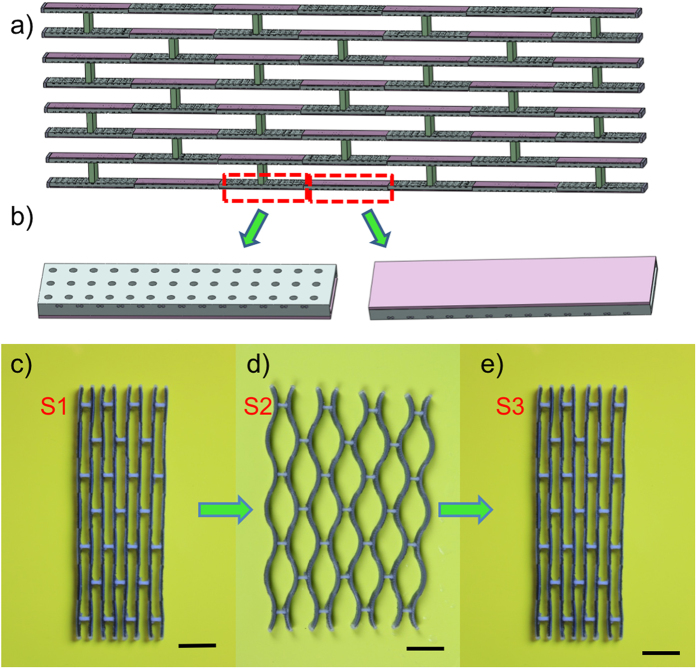
Self-folding and unfolding of a structure with periodic macro-structures. (**a**) The schematic graph of the two-way activation periodic structure. (**b**) The details of the two-way activation design. (**c–e**) Experimental results. The scale bars in (**c–e**) are 15 mm.

Metamaterials are rationally designed artificial materials that gain their properties from structure rather than composition. They have been explored for properties that have not yet been found in nature, such as photonic^34^, negative Poisson’s ratio^35^, acoustic^36^ and mechanical systems^37^. Metamaterials usually achieve their unprecedented properties by designing periodic structures at different length scales. Here, we demonstrate a periodic structure that can be reversibly switched between two configurations. Figure 8a,b show the schematic graphs of the design, where typical reversible shape-changing cells are organized opposite to each other at constant intervals. In each cell, the thicknesses of the elastomer, the hydrogel, and the SMP layers were 0.3 mm, 0.5 mm and 0.2 mm, respectively. The printed structure is shown in Fig. 8c. When under the activation, the elements bent in the opposite direction, leading to a straight-line change to an S-shaped line (Fig. 8d). The structure expanded in one direction while it shrank in another direction; the level of shrinking was determined by the dimensions of the elements, not by the intrinsic Poisson ratio of the constituent materials. Similarly, the structure could recover its initial state by thermal activation as shown in Fig. 8e.

### 3D shape change objects

**Figure 9:**
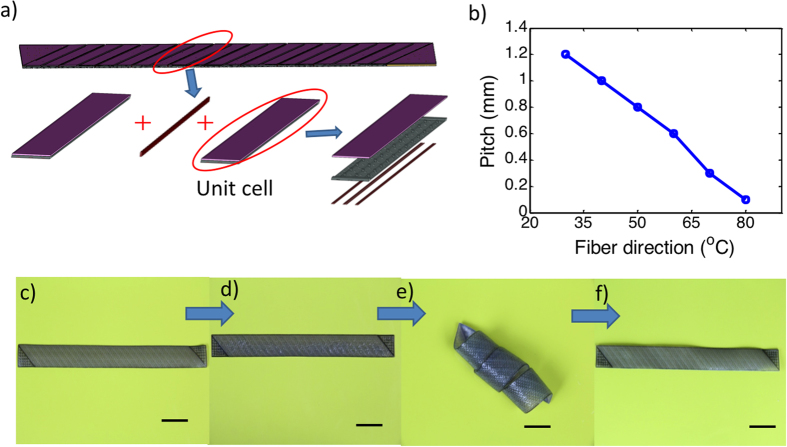
A self coiling and uncoiling strip. (**a**) The schematic graph of the design; (**b**) pitch size as a function of fiber direction. (**c–f**) Images of the samples during the coiling and uncoiling process. The scale bars represent 20 mm.

Controllable self-shaping helical strips have been of increasing research interest due to their potential use in applications ranging from drug delivery systems to biological force probes^38^. By applying the proposed reversible actuation design, a self-coiling/uncoiling helical configuration was realized. Figure 9a shows the schematic graph of the design. Here, we used reinforcing fibers to realize anisotropic mechanical behavior of the structure, which would lead to helical curling. Specifically, the SMP fibers were placed between unit cells and also the bottom of the elastomer layer to induce the anisotropic property of the strip. The general element was designed with thicknesses of the elastomer, the hydrogel, and the SMP to be 0.13 mm, 0.25 mm, and 0.22 mm, respectively. Here, an overall thinner structure was used in order to enable both bending and curling.

Figure 9c shows the printed strip. After being immersed in low temperature water (3 °C) for 12 hrs, the oriented strip had very little bending as shown in 9d. When being putting in high temperature water, a helical configuration was obtained (Fig. 9e). The pitch of the helical configuration depended on the fiber direction (Fig. 9b). As shown in Fig. 9b, when fiber direction increases, the pitch of the structure decreases linearly, which has been discussed elsewhere^39^,^40^. After drying in a cold condition, the helical configuration was fixed and nearly no change took place. When being put in high temperature water again, the helical structure became flat completely under thermal activation (Fig. 9f).

### Reversible folding/unfolding origami

**Figure 10:**
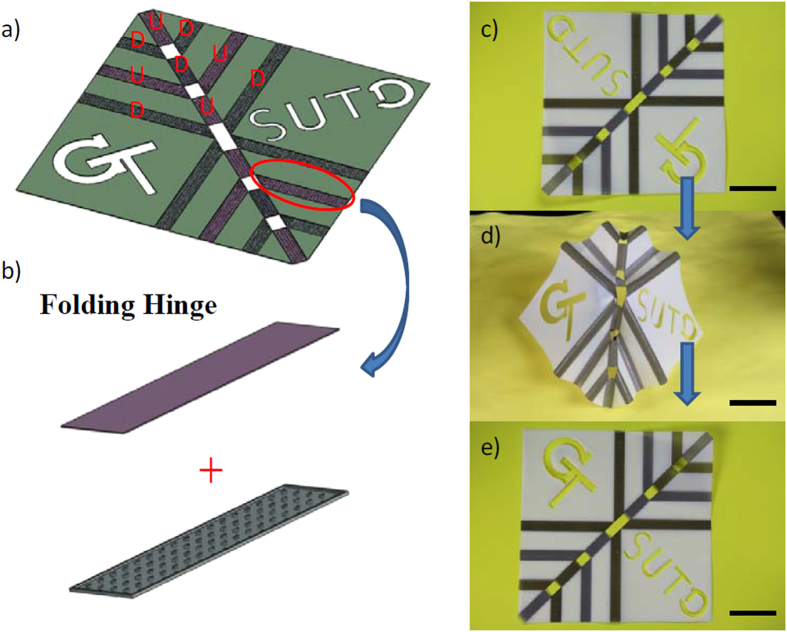
Self-folding/unfolding origami. (**a**) A schematic graph of the design; (**b**) the hinge uses the reversible actuation element. (**c–e**) The reversible actuation of the sheet changing from flat sheet to folded shape, to flat sheet again. The scale bars in (**c–e**) are 45 mm.

Origami folding has attracted great research interest in recent years due to its potential application to active structures. For structural engineers, origami is a rich source of inspiration, and it has found its way into a wide range of structural applications, ranging from wrapping solar cells to medical stents to emergency shelters. Figure 10a shows an origami design by Fuchi *et al*.^41^, who developed a design methodology for origami folding actuators. Following their work, an origami actuator was designed by using reversible components. Here the hinge element had the thicknesses of 0.25 mm, 0.4 mm and 0.15 mm for the elastomer, the hydrogel and the SMP layers, respectively. In Fig. 10a, U and D indicate the bending directions of the hinge with U meaning upward and D for downward, respectively. To realize the desired origami folding, we printed the panels by Verowhite materials connected by designed hinges. Due to the difference in bending stiffness, bending along the short edge requires much lower bending energy, thus is the preferred bending direction. We have demonstrated this by a simple theoretical model in S5 of Supplementary Materials. The as-printed sheet is shown in Fig. 10c. After being put in cold water for 10 hrs then transferred to hot water, the plane sheet folded into a 3D origami structure as shown in Fig. 10d. After being sufficiently dried in cold air and being heated by hot water, the sheet recovered its initial flat configuration (Fig. 10e).

**Figure 11:**
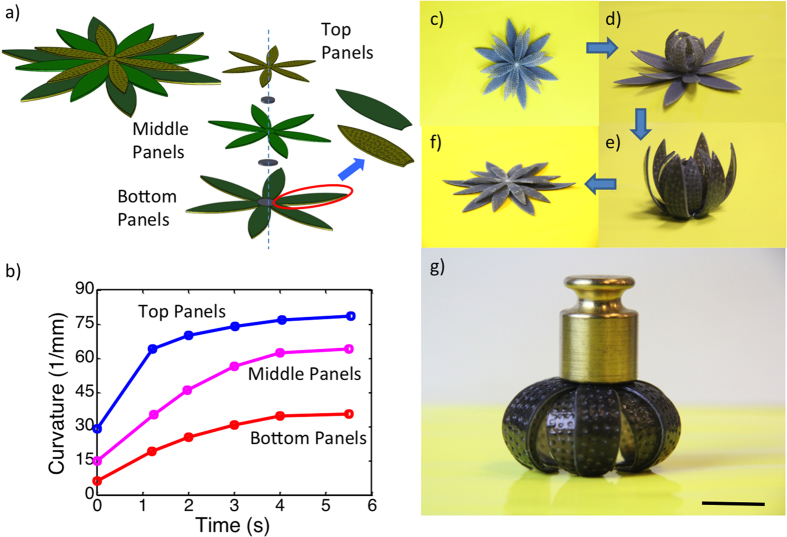
A self-folding/unfolding flower. (**a**) Schematic of two activated shape memory petal-like structure. (**b**) The curvature change under thermal activation as functions of time. (**c–f**) The sequence of reversible actuation. (**g**) The dried configuration is stiff and can carry a load of 25 g. The scale bar in (**g**) is 12.5 mm.

As a final example, we demonstrate a lotus flower. We designed and printed a flower-shaped 3D structure composed of three types of petals, and experimentally demonstrated the control of folding of petals as a function of controllable geometrical size. According to the discussion in Fig. 5, for designs with the same total and elastomer layer thicknesses, those with thinner SMP layers promote larger curvatures and faster response speeds. By elaborating the design of geometrical size, we can create the sequence folding of petals like a flower. The three layers were designed with different thickness ratios of layers of the elastomer, the hydrogel, and the SMP as shown in Fig. 11a. The inner layer was designed with the thicknesses of the elastomer, the hydrogel and the SMP layers as 0.2 mm, 0.4 mm, and 0.3 mm. The second layer was designed with the thicknesses of 0.2 mm, 0.3 mm, and 0.4 mm for the elastomer, the hydrogel and the SMP layers; and the outer layer was designed with the thicknesses of the elastomer, hydrogel and SMP layers as 0.2 mm, 0.2 mm, and 0.5 mm. The corresponding curvature change profiles were characterized by three different speeds for three layers as shown in Fig. 11b. After putting this structure in low temperature water for 12 hrs, the inner layer of petals bent a little as shown in Fig. 11c. The structure was then immersed in high temperature water. Immediately, all the layers bent, forming a flower-like configuration (Fig. 11d,e). Taking the structure out of hot water and letting it dry, the structure maintained the flower shape and was stiff. As shown in Fig. 11g, it could carry a load of 25 g. The flower-like structure was then put into hot water and the structure became flat again (Fig. 11f). This process can be repeated many times.

## Discussion

Designing structures with large reversible shape changes is highly desirable for many engineering and biomedical applications. However, the materials available for large and reversible shape change are rare. Hydrogels are one of the most explored materials, but they are typically soft, with shear modulus typically in the range of a few to a few tens to hundreds of kPa. In this paper, we demonstrate by combining SMPs with hydrogels that we are able to achieve the reversible shape change of structures with relatively complicated geometries. It should be noted that although in principle the proposed design does not rely on 3D printing, the great manufacturing flexibility of 3D printing for complicated geometries is essential to the successful realization of the design, as the designs in this work require complicated layout of different materials at micrometers length scales, which are, if not impossible, extremely difficult to implement.

In the current design, shape change occurs within ~10 s when the structure is immersed into hot water. However, a whole reversible actuation cycle takes ~10–20 hrs, where the majority of the time is on hydrogel swelling and shrinking due to water intake and outtake. The total time can be significantly reduced to a few hours if environmentally responsive hydrogels can be used in place of hydrogels and to even a few minutes if the dimensions of the component can be reduced to micrometer size. Although our current 3D printer does not allow such hydrogels to be printed, we do expect they will be available in the near future.

In the current approach, hot water is used to heat the structure. However, we envision other heating methods, such as Joule heating by electrical current, or electromagnetic field can be used to achieve the same effects. With the current fast development of hybrid 3D printing technology, we fully expect that this can be achieved through one design process.

## Methods

### Material and experiments

The materials used in this study were digital materials that are available from the multimaterial 3D printer (Objet Connex 260, Stratasys, Edina, MN, USA). The so-called digital materials refer to those created through mixing two base model materials, i.e., VeroWhite and Tangoblack, by jet spaying before UV curing. Verowhite is a rigid plastic at room temperature polymerized with ink containing isobornyl acrylate, acrylic monomer, urethane acrylate, epoxy acrylate, acrylic monomer, acrylic oligomer, and photo-initiators. Tangoblack is a rubbery material at room temperature polymerized by monomers containing urethane acrylate oligomer, Exo-1,7,7-trimethylbicyclo [2.2.1] hept-2-yl acrylate, methacrylate oligomer, polyurethane resin and photo initiator. The digital materials consist of varying compositions of these two materials that lead to different thermomechanical properties.

**Figure 12:**
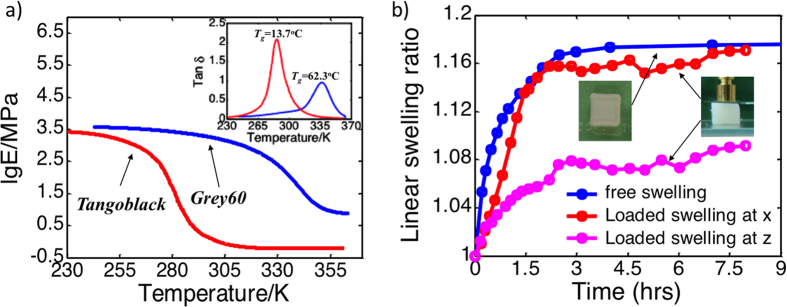
Characterization of printed materials. (**a**) Thermomechanical properties of the printed SMP and the elastomer. (**b**) Swelling behaviors of the hydrogel.

In this work, the thermomechanical properties of digital materials Grey60 and Tangoblack were used. After the samples were printed with the dimension of 15 mm (height) × 3 mm (width) × 0.6 mm (thickness), thermomechanical properties were characterized by a dynamic mechanical analyzer (DMA, Model Q800, TA Instruments, New Castle, DE, USA). The samples were firstly heated up to 100 °C in the DMA tester and stabilized for 20 minutes to ensure thermal equilibrium, then a preload of 1 kPa was applied. During the DMA test, the strain was oscillated at a frequency of 1 Hz with a peak-to-peak amplitude of 0.1% while the temperature was decreased from 100 °C to a given low temperature at a rate of 2 °C/min. Figure 12a shows tanδ as functions of temperature. The temperature corresponding to the peak of the tanδ curve is taken to be the glass transition temperature *T*_*g*_^42^,^43^. The Tg for Grey60 is 48 °C, and is −5 °C for TB.

### Hydrogel

The hydrogel swells when immersing in water. Two types of experiments, i.e., free swelling experiment and confined swelling experiment, were conducted on hydrogels as shown in the inset of Fig. 12b. For the free swelling experiment, isotropic expansion of samples can be observed, with a maximum linear swelling ratio of 1.18 at room temperature (25 °C). When put in the water with a weight of 2.0 g on top of the sample in confined swelling experiment, an anisotropic swelling property was observed, with a small linear swelling ratio of 1.07 along thickness direction, and a linear swelling ratio of 1.16 along the direction without confinement. Figure 12b also shows the swelling ratios as a function of time for the cases of free swelling and constrained swelling.

### FEM Models

FEM simulations were carried out to study the reversible actuation of the structure. To simplify the model, only one cell was considered, with three layers and one diffusion hole in the elastomer layer (as shown in Fig. 3a). To further simplify, a quarter of this cell was modelled and the symmetrical boundary conditions were applied on the *x*-positive surface and the *y*-positive surface. The *x*-negative surface and *y*-negative surface were allowed to move freely in the *x*- and *y*-directly, respectively, but constraints were applied so that they maintained a flat surface during the deformation. The top and bottom z-surfaces were allowed to move freely. A chemical potential boundary condition was applied in the hole area of the hydrogel surface. The hydrogel was modelled by a user element built by Chester^33^, and the hybrid thermal-mechanical elements C3D8HT were applied for the elastomer and the SMP layers. The simplified FEM model consisted of 7165 elements.

### Constitutive relations for the SMP and the hydrogel

The applied constitutive models for the SMP and the hydrogel are presented in the supplementary material.

## Additional Information

**How to cite this article**: Mao, Y. *et al*. 3D Printed Reversible Shape Changing Components with Stimuli Responsive Materials. *Sci. Rep.* **6**, 24761; doi: 10.1038/srep24761 (2016).

## Supplementary Material

Supplementary Information

## Figures and Tables

**Table 1:** Geometrical size effect on bending angle.

Samplenumber	SMP(mm)	Hydrogel(mm)	Elastomer(mm)	Bending angle	
				Low temperature(3 °C)	High temperature(75 °C)
1	0.5	1	0.5	13.5	144.6
2	0.5	1	1	9.2	101.4
3	0.5	1.5	0.5	10.7	125.3
4	0.5	1.5	1	6.9	93.4
5	0.5	2	0.5	8.3	95.5
6	0.5	2	1	8.7	93.1
