# Supplementary material for: 3D Printed Reversible Shape Changing Components with Stimuli Responsive Materials
Source: Sci Rep. 2016 Apr 25;6:24761. doi: 10.1038/srep24761 (PMC4842966; doi:10.1038/srep24761)
Supplement: Supplementary Information [file srep24761-s1.pdf]

Supplementary Materials

3D Printed Reversible Shape Changing Components with Stimuli Responsive Materials

Yiqi Mao[1§], Zhen Ding[2§], Chao Yuan[3], Shigang Ai[4], Michael Isakov[1], Jiangtao Wu[1], Tiejun Wang[3], Martin L. Dunn[2*], H. Jerry Qi[1*]

[1]The George W. Woodruff School of Mechanical Engineering, Georgia Institute of Technology, Atlanta, GA 30332, USA

[2]Singapore University of Technology and Design, Singapore 138682, Singapore

[3]School of Aerospace Science, Xian Jiaotong University, Xian 710049, China

[4]Department of Mechanics, School of Civil Engineering, Beijing JiaoTong University, Beijing, 100044, China

Corresponding authors: Prof. H. Jerry Qi, qih@me.gatech.edu; Prof. Martin L. Dunn, martin_dunn@sutd.edu.sg

§ These authors contribute equally to this paper.

S1. The Reversible actuation at different temperatures

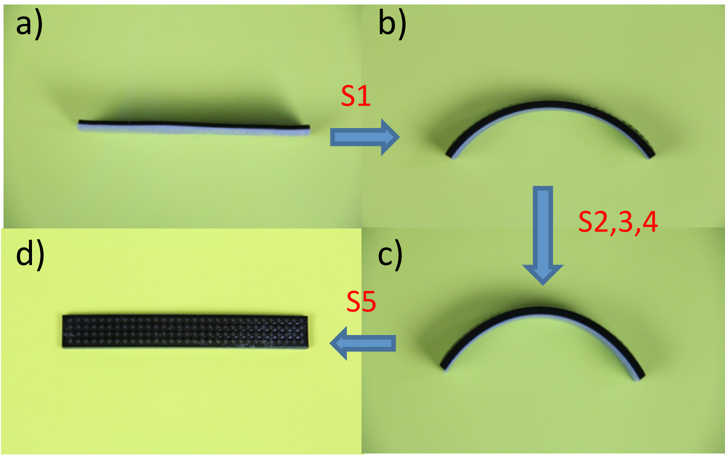

Figure S1. Bending of the reversible strip. a) The printed strip after the first cycle of actuation; b) it bends relatively large after immersing in room temperature water for 12hrs; c) it bends more when immersing in hot water; d) after drying in low temperature air and then putting in the high temperature water, it returns to the straight shape.

The strip, after the first cycle of actuation (Fig. S1a), was immersed in RT water (25°C). After 12hrs, the sample bent (S1, Fig. S1b), with a bending angle that is larger than that in cold water. This is because the SMP has a lower modulus at the room temperature than that in cold water. When the sample was immersed in high temperature water (75°C) again, it showed a nearly identical bending angle as that in the first cycle (Fig. S1c). After drying in low temperature, the sample completely recovered into its initial state (Fig. S1d).

S2. Actuation in oven

Fig. S2 shows the bending angle as functions of heating time in water and in oven in step S2, respectively. It can be seen that the same bending angle can be achieved in oven as compared to that in water, indicating there is no (or very little) water in-taking during the fast bending of the strip at high temperature. Bending in oven, however, is slower, as the connective heating by air is much slower than the conductive heating by water when the strip is immersed in hot water.

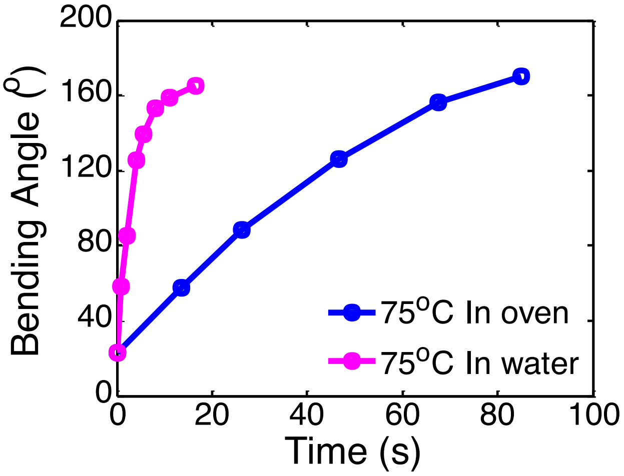

Figure S2. Bending angle as functions of time for heating in water and oven in step S2, respectively.

S.3Constitutive models for the hydrogel and the SMP

The non-equilibrium swelling model for the hydrogel and the thermomechanical model for the SMP are given below, respectively.

S3.1*Thermo-chemo-mechanicalproperty of hydrogel*

The constitutive model for the hydrogel follows the previous work[1,2], and is briefly presented here. For the fluid flux, the spatial fluid flux, $\mathbf{j}$, depends linearly on the spatial gradient of the chemical potential, $\mathrm{grad}\mu$, with the mobility tensor taken to be isotropic so that

$$\mathbf{j} = -m\,\mathrm{grad}\mu, \quad \text{(S1)}$$

where m is a scalar mobility coefficient, which in general is an isotropic function of the stretch and the fluid concentration.

The local balance for the fluid concentration is

$$\dot{c}_R = -J\mathrm{div}\mathbf{j}, \quad \text{(S2)}$$

Assume swelling stretch is

$$\lambda^s = \left(1+\Omega c_R\right)^{1/3}, \quad \text{(S3)}$$

where c_R represents the fluid concentration measured in moles of fluid per unit reference volume of the dry material.

Then the constitutive equation for the Cauchy stress is given as

$$\mathbf{T} = J^{-1}\left(G\left(\mathbf{B}-\mathbf{I}\right)+J^{s}K\left(\ln J^{e}\right)\mathbf{I}\right), \tag{S4}$$

And the chemical potential is given by

$$\mu = \mu^{0} + R\theta\left(\ln\left(1-\phi\right)+\phi+\chi\phi^{2}\right)-\Omega K\left(\ln J^{e}\right)+\frac{1}{2}K\Omega\left(\ln J^{e}\right). \tag{S5}$$

S3.2*Thermomechanical model of She SMP*

The multi-branch model (also referred as generalized standard linear solid model) has been shown to be able to capture the shape memory effects of the polymers[3]. Figure S3 shows a schematic representation of the model, where the choice of the number of branches depends on the width of glass transition and the structure of the polymers. In current analysis, 15 non-equilibrium branches and 1 equilibrium branch are adopted.

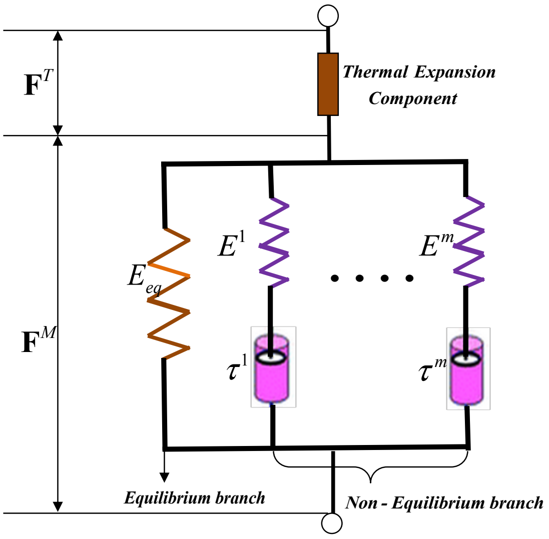

FigureS3.1D rheological representain of the multi-branch model.

The total deformation gradient of the model $\mathbf{F}$ is decomposed as:

$$\mathbf{F} = \mathbf{F}_M\mathbf{F}_T, \tag{S6}$$

where $\mathbf{F}_M$ is the mechanical deformation gradient and $\mathbf{F}_T$ is the thermal deformation gradient.

The total Cauchy stress of the model $\boldsymbol{\sigma}$ is given as

$$\boldsymbol{\sigma} = \boldsymbol{\sigma}_{eq} + \sum_{i=1}^{m} \boldsymbol{\sigma}^{i}, \tag{S7}$$

where $\boldsymbol{\sigma}_{eq}$ and $\boldsymbol{\sigma}^{i}$ are the Cauchy stresses in the equilibrium and the ith ($1 \le i \le m$)

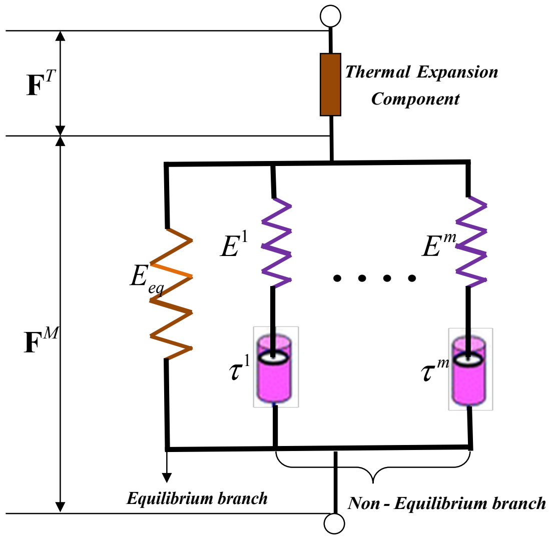

nonequilibrium branches (as shown in FigureS), respectively.

(a) Thermal Expansion

The thermal expansion or contraction of the constructed thermal component in the model is assumed to be isotropic, i.e.

$$\mathbf{F}_T = J_T \mathbf{I}, \tag{S8}$$

where $\mathbf{I}$ is the second order unit tensor. J_T is the volume change due to thermal expansion/contraction and is defined as:

$$J_T = \frac{V(T,t)}{V_0} = \left[1 + 3\alpha_r (T - T_0)\right](1+\delta), \tag{S9}$$

where $V(T,t)$ is the volume at time t and temperature T. V_0 is the reference volume at the reference temperature T_0. α_r is the linear coefficient of thermal expansion (CTE) in the rubbery state and δ characterizes the deviation of volume from equilibrium volume and

$$\delta = \frac{V(T,t)}{V_{eq}(T)} - 1, \quad V_{eq}(T) = \left[1 + 3\alpha_r (T - T_0)\right] V_0, \tag{S10}$$

where$d$ is calculated by the well-known KAHR 33-parameter.

(b) Equilibrium Branch

The Cauchy stress tensor in the equilibrium branch uses Arruda-Boyce eight-chain model, i.e.

$$\boldsymbol{\sigma}_{\mathrm{eq}} = \frac{nk_B T}{3J_{\mathrm{M}}} \frac{\sqrt{N}}{\lambda_{chain}} \mathcal{L}^{-1}\left(\frac{\lambda_{chain}}{\sqrt{N}}\right) \overline{\mathbf{B}}' + K\left(J_{\mathrm{M}} - 1\right)\mathbf{I},$$

$$\overline{\mathbf{B}}' = \overline{\mathbf{B}} - 1/3\,\mathrm{tr}\left(\overline{\mathbf{B}}\right)\mathbf{I}, \quad \overline{\mathbf{B}} = \overline{\mathbf{F}_{\mathrm{M}}}\,\overline{\mathbf{F}_{\mathrm{M}}}^{\mathrm{T}}, \quad \overline{\mathbf{F}_{\mathrm{M}}} = J_{\mathrm{M}}^{-1/3}\mathbf{F}_{\mathrm{M}}, \tag{S11}$$

$$\lambda_{chain} = \sqrt{tr\left(\overline{\mathbf{B}}\right)/3}, \mathcal{L}(\beta) = \coth\beta - 1/\beta.$$

where$n$ is the crosslinking density, k_B is Boltzmann's constant, T is the temperature, N is the number of Kuhn segments between two crosslink sites (and/or strong physical entanglements). The temperature dependent shear modulus $\mu_r(T)$ of the elastomer in the equilibriumstate (which is an indication of entropic elasticity) is given by $nk_B T$. K is the bulk modulus and is typically orders of magnitude larger than μ_r to ensure material incompressibility.

(c) Non-equilibrium Branch

We attempted a unified viscous flow rule for all non-equilibrium branches, and the relaxation time is considered as a function of temperatures for each branch. For the *i-th*nonequilibrium branch ($1 \le i \le m$), the deformation gradient can be further decomposed into an elastic part and a viscous part

$$\mathbf{F}_M^i = \mathbf{F}_e^i \mathbf{F}_v^i, \tag{S12}$$

where$\mathbf{F}_v^i$ is a relaxed configuration obtained by elastically unloading by $\mathbf{F}_e^i$. The Cauchy stress can be calculated using $\mathbf{F}_e^i$,

$$\boldsymbol{\sigma}^i = \frac{1}{J_e^i}\left[\mathbf{L}_e^i(T):\mathbf{E}_e^i\right], \text{ for } 1 \le i \le m, \tag{S13a}$$

$$J_e^i = \det\left(\mathbf{F}_e^i\right), \ \mathbf{E}_e^i = \ln \mathbf{V}_e^i, \ \mathbf{V}_e^i = \mathbf{F}_e^i \mathbf{R}_e^{iT}, \tag{S13b}$$

and $\mathbf{L}_e^i(T)$ is the fourth order isotropic elasticity tensor in the i-thnonequilibrium branch ($1 \le i \le m$), which is taken to be temperature independent in general, i.e.

$$\mathbf{L}_e^i(T) = 2G^i\left(\mathbf{I} - \frac{1}{3}\mathbf{I}\otimes\mathbf{I}\right) + K^i\mathbf{I}\otimes\mathbf{I}, \text{ for } 1 \le i \le m, \tag{S14}$$

where$\mathbf{I}$ is the fourth order identity tensor, G^i and K^i are shear and bulk moduli for each nonequilibrium branch ($1 \le i \le m$), respectively.

For the rnonequilibrium branches ($1 \le i \le m$), it is assumed that all the rubbery branches have the same shear modulus, i.e.

$$G^i(T) = n_R k_B T \text{ for } 1 \le i \le m, \tag{S15}$$

where n_R is the crosslinking density. Then The elastic modulus in each nonequilibrium branch is calculated as

$$E^i(T) = \frac{G^i(T)}{2(1+v^i)} \text{ for } 1 \le i \le m, \tag{S16}$$

In the nonequilibrium branches, the temperature dependent relaxation times are calculated according to the thermorheological simplicity principle,

$$\tau^i(T) = \tau_0^i \alpha_T(T) \text{ for } 1 \le i \le m, \tag{S17}$$

where$\alpha_T(T)$is the time-temperature superposition (TTSP) shift factor and τ_0^i is the relaxation time at the reference temperature when $\alpha_T(T) = 1$. At temperatures around or above T_s, the WLF equation is applied,

$$\log \alpha_T(T) = -\frac{C_1\left(T - T_M\right)}{C_2 + \left(T - T_M\right)}, \tag{S18}$$

whereC_1 and C_2 are material constants and T_M is the WLF reference temperature.

When the temperature is below T_s, $\alpha_T(T)$ follows the Arrhenius-type behavior:

	Description	*Value*
G	Shear modulus	0.095 MPa
K	Volume modulus	100 MPa
Ω	Mole volume	1.0×10^{-4} m^3/mol
χ	Diffusion parameter	0.1
μ^o	Initial chemical	0.0 J/mol
D	Diffusion coefficient	5.8×10^{-8} m^2/s

$$\ln\alpha_T(T) = -\frac{AF_c}{k_b}\left(\frac{1}{T}-\frac{1}{T_g}\right), \tag{S19}$$

where A and Fc are material constants, kb is Boltzman's constant. Here, Ts is calculated by equating $\alpha_T(T)$ in Eqs. **Error! Reference source not found.** and **Error! Reference source not found.**.

S4.Material parameters

(a) Material Parameters for the hydrogel model

Table S1 Mechanical and diffusion parameters for hydrogel

Hydrogel sampleswere printed with the dimension of 15mm (height)×3mm(width)×0.6mm(thickness), and then were stretched to break under displacement control by a dynamic mechanical analyzer (DMA, Model Q800, TA Instruments, New Castle, DE, USA). The obtained stress-strain curve is shown in Fig.S4, where the fitted simulation results is also presented.The fitted material parameters are listed in Table S1.

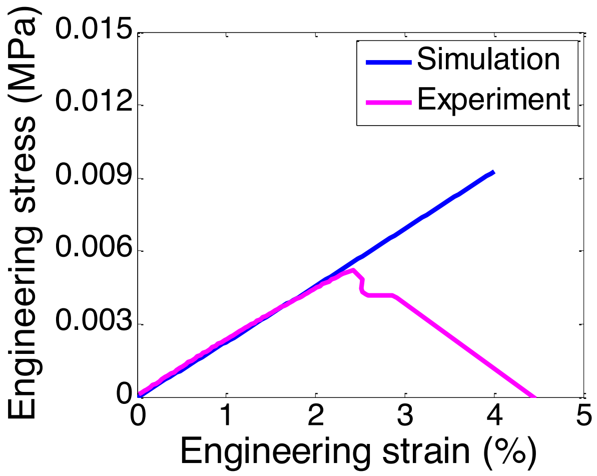

Figure S4. Engineering stress-engineering strain curves of hydrogel.

To obtain the material parameters associated with diffusion, two swelling experiments were conducted: one free swelling and one with a deadweight of 2g. By coupling the diffusion equation and chemical potential equation, the diffusion parameters are calibrated by one diffusion model and listed in Table S1. The fitted free swelling and weighted swelling ratio along with diffusion time are listed in Fig. S5.

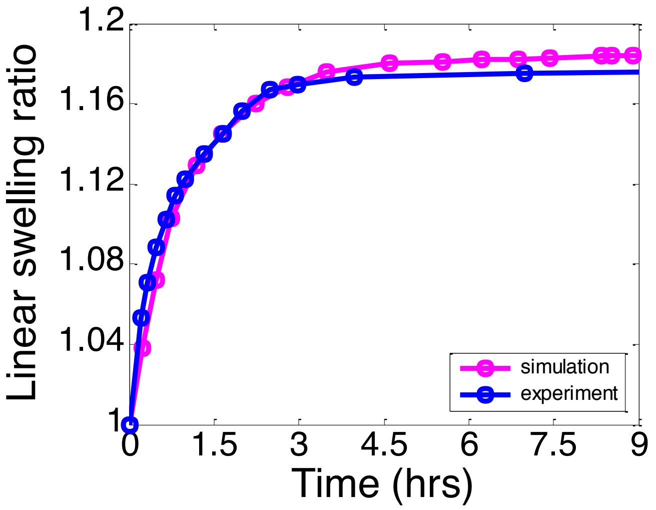

(a)

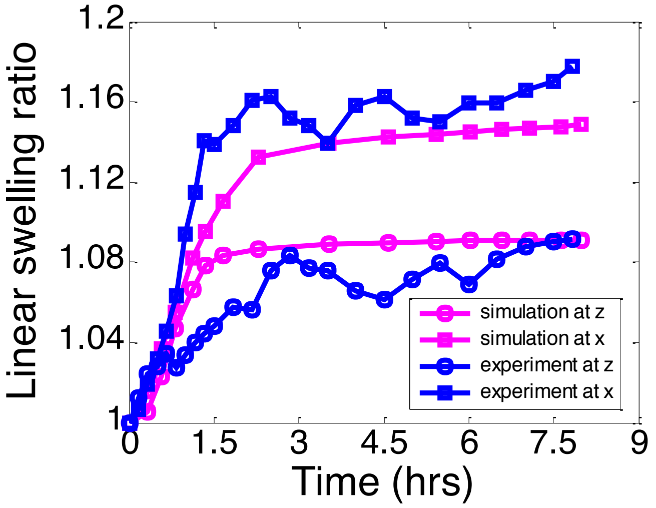

(b)

Figure S5.Swelling ratio of hydrogel (a) without weight and (b) with a 2g weight.

(b) Material parameters forthe SMP model

Material parameters for the SMP and the elastomerwere obtained by fitting with experimental results. The method to calibrate corresponding material parameters for all equilibrium and non-equilibrium branches follows our previous work[4]. The fitted storage modulus and $\tan\delta$ are presented and compared with the experiment in Fig.S6. All the material parameters are listed in Table S2.

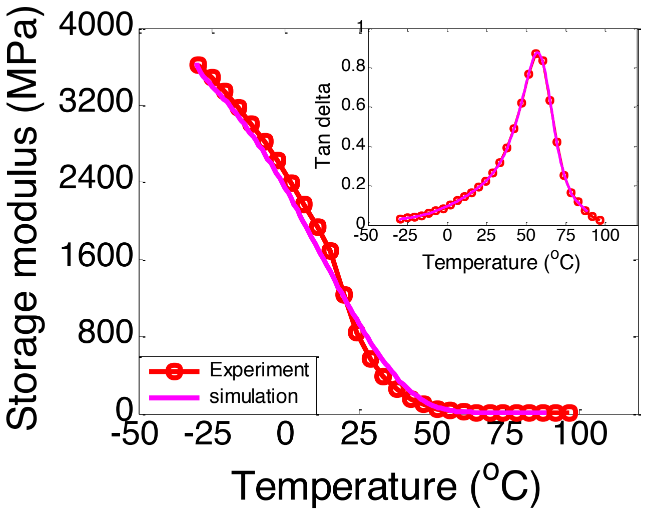

(a)

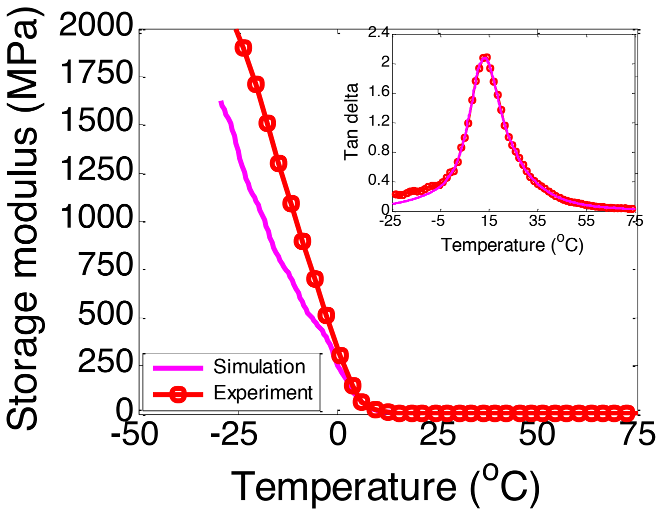

(b)

Figure S6. Fitted storage modulus and tanδfor (a) the SMP and (b) The elastomer.

Table S2.Material parameters for the SMP and the elastomer.

	Es (MPa)	*AFKB*	*C_1*	*C_2*	*T_g (K)*	*T_m*
The SMP	5.2	-10000	12.6	47.6	327.6	308.5
The elastomer	0.6	-20500	12.4	51.6	286.7	271.0

The SMP	$E^1 \sim E^6$ *(MPa)*	2.0e3	2.1e3	6.0e2	2.7e2	3.2e2	2.1e2
	$\tau_0^1 \sim \tau_0^6$ *(s)*	1.2e-5	1.0e-4	6.0e-3	1.0e-2	1.1e-1	1.0
	$E^7 \sim E^{12}$ *(MPa)*	1.3e2	7.8e1	4.1e1	1.7e1	6.2	1.5
	$\tau_0^7 \sim \tau_0^{12}$ *(s)*	9.7	7.9e2	5.8e2	3.8e3	2.5e4	2.0e5
	$E^{13} \sim E^{15}$ *(MPa)*	1.1e-1	4.0e-3	1.0e-3			
	$\tau_0^{13} \sim \tau_0^{15}$ *(s)*	2.8e6	2.0e7	2.0e8			
The elastomer	$E^1 \sim E^6$ *(MPa)*	3.3e2	3.2e2	3.8e2	4.5e2	3.6e2	2.8e2
	$\tau_0^1 \sim \tau_0^6$ *(s)*	2.0e-7	6.e-6	8.e-5	9.4e-4	1.0e-2	9.8e-2
	$E^7 \sim E^{12}$ *(MPa)*	2.4e2	1.6e2	6.4e1	1.0e1	2.0	7.8e-1
	$\tau_0^7 \sim \tau_0^{12}$ *(s)*	1	1.0e1	6.4e1	3.6e2	2.4e3	2.0e4
	$E^{13} \sim E^{15}$ *(MPa)*	2.4e-1	6.7e-2	2.7e-2			
	$\tau_0^{13} \sim \tau_0^{15}$ *(s)*	2.0e5	2.0e6	2.4e7			

S5. Theoretical analysis on folding angle of the hinge in origami design

In this self-folding origami design, the hinge is composed of SMP, Gel and Tangoblack, while the panel is printed by Verowhite, a very stiff digital material with very high modulus. Then it is not difficult to find the bending moment of inertia along the long edge is largely greater than that along the short edge. To address this more clearly, we give a simple calculation on the bending angle in the following,

From classical material mechanics, the bending angle of a beam is calculated by

$$\theta = \frac{ML}{EI} \tag{S20}$$

here θ is the bending angle, M is the moment, EI is the bending stiffness and L is the length of the beam.

We simplify the current problem to calculate bending angle of representative element, a rectangular square marked by red dashed line, along the x direction and y direction as shown in the Fig.S7. The element has a length of L, and the hinge has a width of w. The thickness of the sheet is h. Assume the generated isotropic swelling force by the hinge is f per unit length, i.e., it is the same along the x- and y-directions.

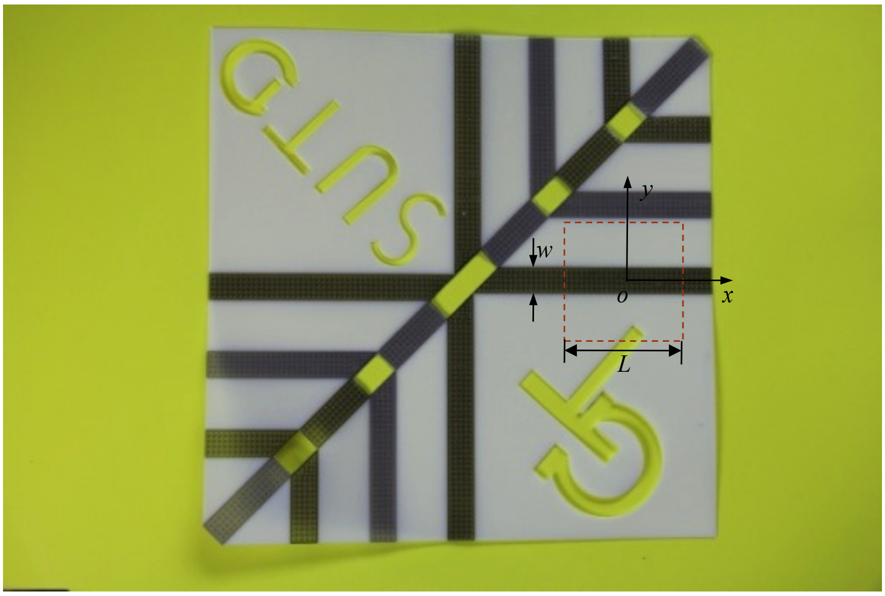

Fig. S7. Folding origami design.

As for the bending along the x-direction (long edge), we have

$$M_x = fLw, L_x = L, EI_x = \frac{\left[E_v(L-w)+E_h w\right]h^3}{12}, \quad \theta_x = \frac{fLw \cdot L}{\frac{\left[E_v(L-w)+E_h w\right]h^3}{12}} \tag{S21}$$

As for the bending along the y direction(short edge), we have

$$M_y = fwL, L_y = w, EI_y = \frac{E_h w h^3}{12}, \theta_y = (fLw \cdot w) \Big/ \left(\frac{E_h w h^3}{12}\right) \tag{S22}$$

Then we have

$$\frac{\theta_y}{\theta_x}=\frac{fLw\cdot w}{E_h wh^3}\cdot\frac{\left[E_v\left(L-w\right)+E_h w\right]h^3}{fLw\cdot L}=\frac{E_v}{E_h}\left(1-\frac{w}{L}\right)+\frac{w}{L} \quad \text{(S23)}$$

here E_v is the modulus of the Verowhite (white panel), and E_h is the effective modulus of the hinge which is the combination of three layers (SMP, Gel and Tangoblack). From the experiment results, the modulus of the Verowhite, SMP, the Tangoblack and the Gel are around 3700MPa, 2500MPa, 1000MPa, and 0.1MPa in the low temperature (2°C),and around 50MPa, 30MPa, 0.5MPa, and 0.1Mpa high temperature (75°C),respectively. The effective modulus of E_h is calculated as ~100MPa at low temperature, and ~0.5MPa at high temperature (75°C) by following equation,

$$\frac{E_h bh^3}{12}=\left(E_{SMP}I\right)_{SMP}+\left(E_{Gel}I\right)_{Gel}+\left(E_{Tangobleck}I\right)_{Tangobleck} \quad \text{(S24)}$$

Therefore we arrive at

$$E_v/E_h\approx 40\text{~}100. \quad \text{(S25)}$$

From Eq.(S23) and Eq.(S25), we can find it is only when length of representative element L equals the width of the hinge w, the bending angle is the same along the x and y. If the L is greater than w, the bending angle along y direction is greater than that along x direction, meaning the hinge is more likely to bend along the short edge, rather than long edge.

Reference in Supplementary Materials

1 Chester, S. A. & Anand, L. A thermo-mechanically coupled theory for fluid permeation in elastomeric materials: application to thermally responsive gels. *Journal of the Mechanics and Physics of Solids* **59**, 1978-2006 (2011).

2 Chester, S. A., Leo, C. V. D. & Anand, L. A finite element implementation of a coupled diffusion-deformation theory for elastomeric gels. *International Journal of Solids and Strucutres* **52**, 1-18 (2015).

3 Yu, K., Ge, Q. & Qi, H. J. Reduced time as a unified parameter determining fixity and free recovery of shape memory polyers. *Nature communications* **5**, 3066 (2014).

4 Yu, K., McClung, A. J. W., Tangdon, G. P., Baur, J. W. & Qi, H. J. A Thermomechanical Constitutive Model for an Epoxy Based Shape Memory

Polymer and its Parameter Identifications. *Mech Time-Depend Mater* **18**, 453-474 (2014).
